# Objective Evaluation of the Quality of Movement in Daily Life after Stroke

**DOI:** 10.3389/fbioe.2015.00210

**Published:** 2016-01-13

**Authors:** Fokke B. van Meulen, Bart Klaassen, Jeremia Held, Jasper Reenalda, Jaap H. Buurke, Bert-Jan F. van Beijnum, Andreas Luft, Peter H. Veltink

**Affiliations:** ^1^Biomedical Signals and Systems, MIRA – Institute for Biomedical Technology and Technical Medicine, University of Twente, Enschede, Netherlands; ^2^Centre for Telematics and Information Technology, University of Twente, Enschede, Netherlands; ^3^Division of Vascular Neurology and Neurorehabilitation, Department of Neurology, University Hospital of Zurich, Zurich, Switzerland; ^4^Roessingh Research and Development, Roessingh Rehabilitation Hospital, Enschede, Netherlands; ^5^Biomechanical Engineering, MIRA – Institute for Biomedical Technology and Technical Medicine, University of Twente, Enschede, Netherlands

**Keywords:** stroke, rehabilitation, inertial sensing, daily life, data processing, technology assessment

## Abstract

**Background:**

Stroke survivors are commonly left with disabilities that impair activities of daily living. The main objective of their rehabilitation program is to maximize the functional performance at home. However, the actual performance of patients in their home environment is unknown. Therefore, objective evaluation of daily life activities of stroke survivors in their physical interaction with the environment is essential for optimal guidance of rehabilitation therapy. Monitoring daily life movements could be very challenging, as it may result in large amounts of data, without any context. Therefore, suitable metrics are necessary to quantify relevant aspects of movement performance during daily life. The objective of this study is to develop data processing methods, which can be used to process movement data into relevant metrics for the evaluation of intra-patient differences in quality of movements in a daily life setting.

**Methods:**

Based on an iterative requirement process, functional and technical requirements were formulated. These were prioritized resulting in a coherent set of metrics. An activity monitor was developed to give context to captured movement data at home. Finally, the metrics will be demonstrated in two stroke participants during and after their rehabilitation phases.

**Results:**

By using the final set of metrics, quality of movement can be evaluated in a daily life setting. As example to demonstrate potential of presented methods, data of two stroke patients were successfully analyzed. Differences between in-clinic measurements and measurements during daily life are observed by applying the presented metrics and visualization methods. Heel height profiles show intra-patient differences in height, distance, stride profile, and variability between strides during a 10-m walk test in the clinic and walking at home. Differences in distance and stride profile between both feet were larger at home, than in clinic. For the upper extremities, the participant was able to reach further away from the pelvis and cover a larger area.

**Discussion:**

Presented methods can be used for the objective evaluation of intra-patient differences in movement quality between in-clinic and daily life measurements. Any observed progression or deterioration of movement quality could be used to decide on continuing, stopping, or adjusting rehabilitation programs.

## 1 Introduction

Patients who have suffered a stroke are commonly left with disabilities that impair activities of daily living. They are trained to recover adequate control over their movements with the objective to optimize their daily life functional performance (Kollen et al., 2006). In current clinical practice of stroke rehabilitation, the capacity of stroke patients to perform functional tasks is assessed using standardized clinical tests. These tests are done regularly during the entire rehabilitation process to predict functional performance at home (Kollen et al., 2006). While the main objective of the rehabilitation program is to maximize the functional performance at home, the actual performance of patients in their home environment is unknown (Bussmann et al., 2009). Therefore, daily life monitoring of the quality of movement during functional activities of stroke survivors in their physical interaction with the environment is essential for optimal guidance of rehabilitation therapy, which goes beyond established activity monitors.

A system for daily life monitoring and assessment of the quality of movements should be a small wearable system, not be directly visible, not stigmatizing, and contain small and embedded sensors (Bergmann and McGregor, 2011). Wearable sensing systems using inertial sensors are already frequently used for the assessment of daily life activity (Moe-Nilssen, 1998; Bussmann et al., 2009; Gebruers et al., 2010; de los Reyes-Guzmán et al., 2014; Veltink et al., 2014; van Meulen et al., 2015). These types of systems are small and not restricted to a lab environment. We previously developed, via an iterative process, a modular sensor system for quantitative analysis of daily life activities of upper and lower extremity motor function (Klaassen et al., 2014; Paradiso et al., 2014; Veltink et al., 2014). This system can be used to obtain knowledge about quality of movement of stroke patients during in-clinic measurements and performing in a daily life setting.

It should be noted that several challenges remain in performing a quantitative analysis of daily life performance using wearable sensing, compared to clinically assess motor capacity using standardized clinical tests. First, metrics need to be developed that quantify relevant aspects of quality of movement during daily life. Metrics that are used in clinic to describe movements cannot directly be transferred to the evaluation of movements in a daily life setting. For instance, in clinical assessment scales, participants are instructed to reach as far as possible; while in a daily life setting, it might not be necessary to reach that far. A second challenge is the absence of context when measuring movements without any visual reference. During the evaluation of movements in a clinical or lab setting, it is known where and what kind of activities a participant is performing. This information is not available in a daily life setting. Therefore, a method is needed to classify the performed activities from the sensed signals. Finally, during continuous home measurements, a full body inertial sensor system will produce large amounts of movement data. Metrics and visualizations derived from these large amounts of data should be presented in a concise manner; otherwise, the evaluation of all data might be very time consuming for care-professionals.

The objective of this study is to develop data processing methods, which can be used to process movement data into relevant metrics for the evaluation of intra-patient differences in the quality of movements in clinic and in a daily life setting. In a collaborative effort of care-professionals, researchers, and engineers, a requirements analysis on methods to assess daily life quality of movements was performed. Subsequently, methods were developed that also should overcome mentioned challenges in the daily life assessment of movements. These methods include the estimation of metrics that show intra-patient differences in the quality of movements between in-clinic measurements and out-clinic measurements. These metrics will be demonstrated in two stroke participants during and after their rehabilitation phases.

## 2 Materials and Methods

This section is divided into six parts: (1) a requirement analysis, resulting in a coherent set of “must-haves”; (2) a description of the metric development process and the underlying theory, resulting in a coherent set of metrics; (3) a description of the sensor system for the assessment of daily life movements in stroke patients; (4) the development of an activity monitor to provide context to the captured movement data of stroke patients performing activities of daily living; (5) the presentation of vast amounts of movement data to care-professional, and (6) an analysis of intra-patient differences, for two cases, in quality of movements between structured in-clinic and unstructured daily life measurements, determined using the developed metrics.

### 2.1 Requirement Analysis

As part of the European project called INTERACTION (Klaassen et al., 2014; Paradiso et al., 2014; Veltink et al., 2014), a requirement analysis was performed as a basis for the metric development. A questionnaire was sent out to selected care-professionals with a background in stroke rehabilitation in the Netherlands (response: n = 12) and Switzerland (response: n = 4). In addition, an interview in a round table setting was held with selected stroke patients (n = 3) in the Netherlands. Furthermore, a consensus meeting with project team members and advisory board (consisting of stroke patients, care-professionals, health insurance companies, and researchers) was organized. Based on the results from the questionnaire, the interviews, and the consensus meeting, and using the PACT (People, Activities, Contexts, and Technologies) (Huis in ‘t Veld et al., 2010) and FICS (Functionalities, Interaction, Context, and Service) (Jackson, 1997) frameworks, user requirements and technical state of the art developments were translated into functional requirements and technical specifications. These requirements and specifications were divided into two groups, namely for lower extremity and upper extremity. The requirements and specifications were prioritized using the MoSCoW technique (“Must-have,” “Should-have,” “Could-have,” and “Won’t have”) (Clegg and Barker, 1994). These “must-have”-requirements were the basis for the methods development and are listed and labeled in Table 1.

**Table 1:** **“Must-haves” as a result of the requirement analysis^a^**.

	Lower extremity	Inc.^b^		Upper extremity	Inc.^b^
	**Which activities are performed?**			**Which activities are performed?**	
LE1	Standing	Yes	UE1	Reaching	Yes
LE2	Sitting	Yes	UE2	Grasping (with gloves)	Yes
LE3	Walking	Yes			
	**Intensity of walking**			**Intensity of reaching**
LE4	Frequency of activities	Yes	UE3	Frequency of activities	Yes
LE5	Duration of activities	Yes	UE4	Duration of activities	Yes
LE6	Speed	Yes	UE5	Unilateral versus bilateral activities	Yes
LE7	Covered walking distance	Yes			
	**Quantification of balance**			**Quality of movement**
LE8	Step length	No	UE6	Hand position relative to pelvis	Yes
LE9	Relative feet position	No	UE7	Flexion and extension in the elbow	Yes
LE10	Stability during stance	No	UE8	Shoulder abduction	Yes
LE11	Stability during single support	Yes	
LE12	Orientation of the feet	No	
LE13	Center of mass movement, relative to base of support	No	
LE14	Knee flexion and extension	No	
LE15	Dorsal/plantar flexion of the ankle	No	
LE16	Circumduction	No	

*^a^When applicable, metrics will be evaluated for the affected and non-affected side*.

*^b^“Must-haves” that are included in the final set of data processing methods*.

### 2.2 Design of Metrics

An iterative design process was adopted to translate the “must-have”-requirements that are technically feasible into metrics the system must contain. These requirements were continually assessed and revised during the design phase of the project to ensure alignment between technical developments and users’ needs. The design process included focus group sessions and teleconferences between engineers, care-professionals, and researchers.

**Figure 1:**
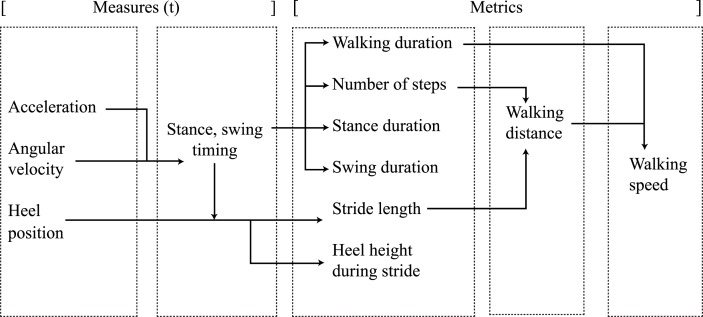
**Lower extremity measures and metric relations**.

**Figure 2:**
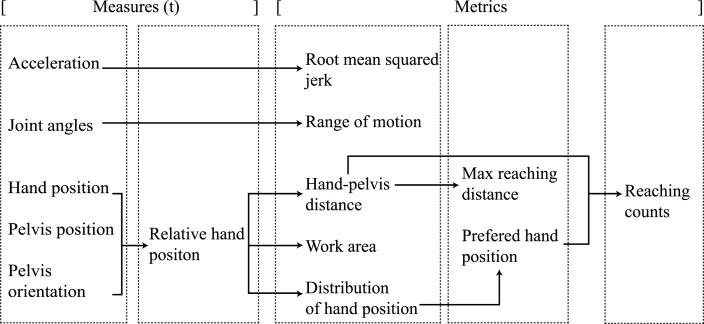
**Upper extremity measures and metric relations**.

In total, 25 focus group sessions and 15 teleconferences were organized between 2014 and 2015, which included discussions and demonstrations of the metrics. Finally, a metrics-overview was created, which includes realizable “must-have” metrics for lower and upper extremities, as shown in Figures 1 and 2, which are further explained in the following sections.

#### 2.2.1 Lower Extremity Metrics

The lower extremity metrics and their relation with system measures (Roetenberg et al., 2009) are shown in Figure 1. All metrics, shown in Figure 1, were realized by evaluating the heel positions and by calculating the timing values of stance and swing during gait, utilizing a step detection algorithm (Skog et al., 2010). Each metric is determined within a selected time window.

The covered requirement *walking distance* (LE7) was directly rendered to the walking distance metric, by combining the number of steps and stride length as shown in Figure 1. Walking distance was included, as it is an important predictor for community walking abilities in stroke patients (Donovan et al., 2008; Fulk et al., 2010). The *walking speed* (LE6) requirement was included, which is regarded as a significant, sensitive, and reliable marker of deficit severity and functional community walking ability and results from the walking distance and walking duration metrics (Dickstein, 2007). The *frequency of activities* (LE4) requirement was translated into the number of steps that a patient takes within a certain time. Furthermore, the *duration of activities* (LE5) requirement was rendered to the walking duration metric.

For quantifying balance during gait, focus was set on *stability during single support* (LE11) requirement, which includes stance and swing times. These classical gait parameters were implemented earlier by, e.g., Kuo and Donelan (2010), who explained the dynamic principles of gait and their clinical implications but also in other studies related to stroke (Von Schroeder et al., 1995; Evans et al., 1997; Haggard and Cockburn, 1998; Bowen et al., 2001; Cockburn et al., 2003; Brach et al., 2008; Balasubramanian et al., 2009; Belda-Lois et al., 2011). Variability in stance and swing time parameters predicts motor disability and, therefore, seems to be related to walking impairments and can be used as a quantifiable biomechanical marker to evaluate motor performance (Brach et al., 2008; Balasubramanian et al., 2009). Due to the limited accuracy of the sensor system (Klaassen et al., 2014) in estimating the *relative feet position* (LE9), *step length* (LE8) requirement was changed into stride length. Studies focusing on fall risks revealed that the stride length, the corresponding variability, and the swing time variability are key parameters for gait characteristics in elderly adults (Hausdorff et al., 2001; Verghese et al., 2009). Stride length is based on the heel position of the foot, an output measure of the applied kinematic model, and the stance and swing timing values. The *center of mass movement* (LE13) and *stability during stance* (LE10) requirements were left-out of the final metric selection, due to the absence of any type of force sensing in the system. Finally, the heel-height profiles were added to the final metric selection, which resulted from discussions in the design process with clinicians. These profiles are important to estimate the risk of falling and show imbalances and impairments during walking (Maki, 1997; Verghese et al., 2009).

#### 2.2.2 Upper Extremity Metrics

The upper extremity metrics target trunk, shoulder, and arm movements. Each of the measures and metrics associated with the upper extremity are shown in Figure 2. The quality of movement requirement *hand position relative to pelvis* (UE6) was rendered to the metrics: hand–pelvis distance, covered work area, and maximum reaching distance for both hands. The hand positions were expressed in a coordinate frame relative to the pelvis orientation and position as described by van Meulen et al. (2015). The relative hand position measure was used to calculate the three-dimensional hand–pelvis distance and work area that patients are able to cover with their hands. In addition, the hand trajectory during a reaching movement was included. Finally, a newly developed metric named “hand distribution” was added as an extra quality of movement requirement, which visualizes the distribution of the patient’s hand position in the transversal plane. The maximum reached distance and the size of the work area, the hand is able to cover, was investigated by van Meulen et al. (2015). They compared these metrics to the upper part of the Fugl–Meyer assessment scale and found a positive correlation. The reaching distance was also mentioned in an earlier study by Zariffa et al. (2012) and was also investigated by Balasubramanian et al. (2012), who provided a definition of reachable work space.

Another requirement is the *flexion and extension in the elbow* (UE7) and the *abduction of the shoulder* (UE8), which was quantified as the joint range of motion metric for the elbow and shoulder. Joint range of motion during functional movements was investigated in several studies (Rönnqvist and Rösblad, 2007; Ellis et al., 2008; Jaspers et al., 2011) and the joint range of motion in complete simulated activities of daily living has also been described extensively in de los Reyes-Guzmán et al. (2014).

A final quality of movement metric was added, related to the smoothness of motion. For each reaching movement of the hand, a smoothness parameter, which is referred in this paper as the root mean squared jerk (RMSJ), is calculated based on the change in acceleration of the hand. The RMSJ, which normalizes the jerk over time, has been applied in several studies, including stroke patients (Young and Marteniuk, 1997; Song et al., 2008; Hogan and Sternad, 2009). The *frequency of activities* (UE3) requirement was translated to a metric showing the number of reaching motions a patient has performed during a selected time period. The number of reaches provides an indication of the overall arm activity level during daily life. Although it is not mentioned in previous research on arm activity of stroke patients (Vega-González and Granat, 2005; de Niet et al., 2007), reaching counts could provide insight in arm activity and, therefore, this is a new metric as well. A reaching activity was derived from the relative hand–pelvis distance and the preferred hand position within a certain measurement time frame during either sitting, standing, or walking.

### 2.3 Sensor System Overview

**Figure 3:**
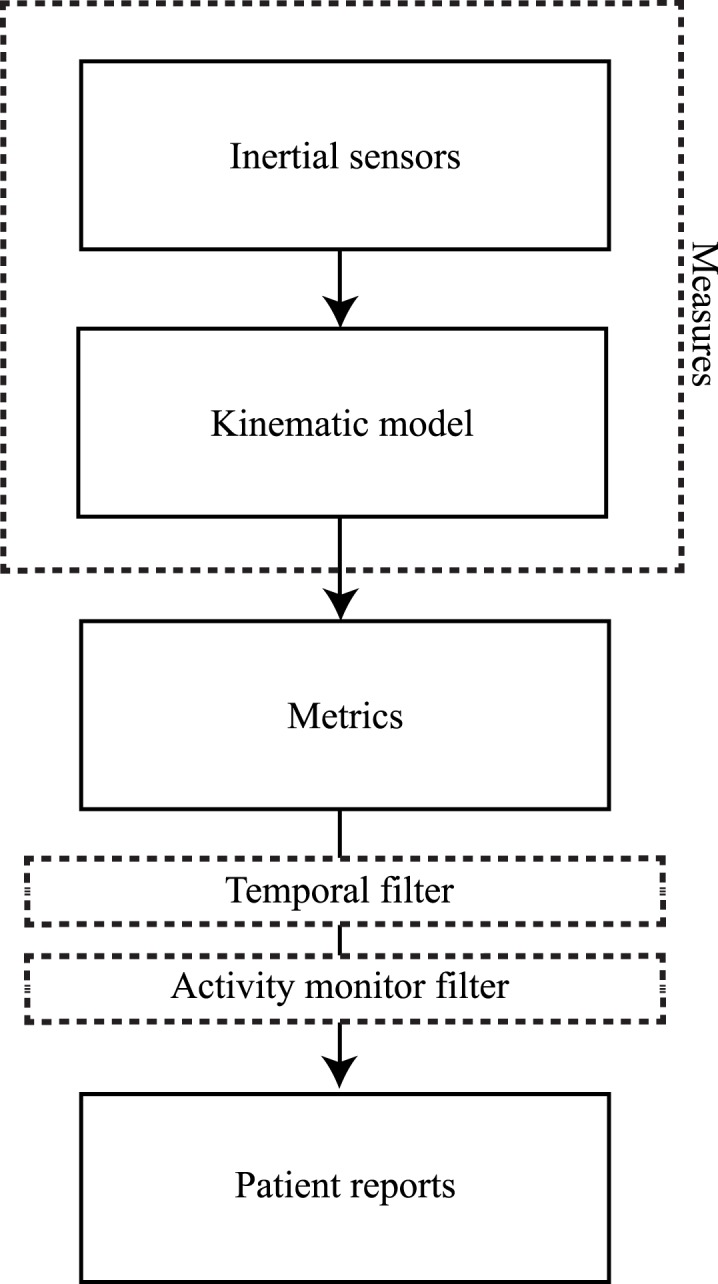
**Data processing steps**. From captured inertial sensor data to patient reports.

Figure 3 shows a flowchart of the main data processing steps. The modular sensor system that was previously developed for the assessment of movements in a daily life setting (Klaassen et al., 2014; Paradiso et al., 2014; Veltink et al., 2014) consists of twelve inertial measurement units (IMUs), each consisting of a three-dimensional accelerometer, gyroscope, and magnetometer. These IMUs are embedded in a garment consisting of a shirt, a pair of shoes, and a pair of trousers (Paradiso et al., 2014). The IMUs are placed on the upper and lower arms, upper and lower legs, both feet, head, pelvis, and sternum. The data are collected at a sampling rate of 20 Hz, which converted from a higher internal sensor sampling rate of 1800 Hz (Klaassen et al., 2014). As part of the Xsens MVN studio software package (Xsens Technologies B.V., Enschede, the Netherlands) (Roetenberg et al., 2009), a full body kinematic model is used to process the captured sensor data and to estimate the three-dimensional body movements over time, including the position and orientation of each body segment as well as the joint angles. From there, selected metrics are computed, which are in turn fed into two filters. The first filter is a temporal selection filter, with which the time period of interest can be selected to analyze the data. The second filter is the activity monitor, which allows the evaluation of certain metrics for specific activities. All data processing steps are performed in an offline environment, using MATLAB^®^ (MathWorks Inc., Natick, MA, USA).

### 2.4 Development of an Activity Monitor

While performing activities of daily living, stroke patients are monitored unobtrusively while wearing a full sensor system. Since visual observation is not employed to monitor the patient, there is a lack of information about the context in which the different activities are performed by the patients. Several metrics are context dependent, such as walking speed or reaching distance. For example, estimating walking speed is only relevant when patients are walking. Another example is the evaluation of reaching distance that should be evaluated without including the arm swing during walking. In order to provide this context to the data, an activity monitor is required. The activity monitor acts as a filter and can be considered a separate step within our data processing methods.

**Figure 4:**
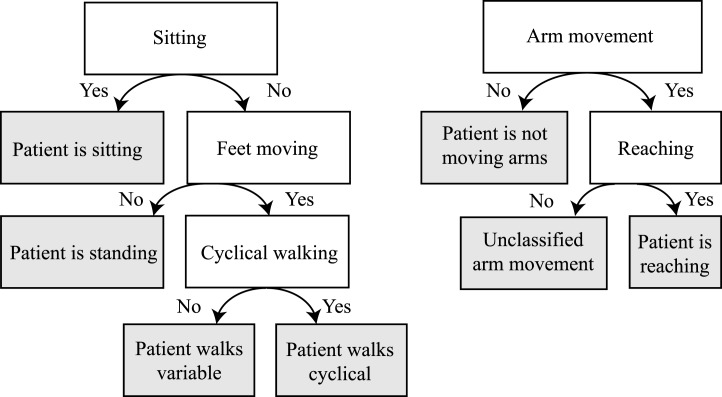
**Decision tree of the activity classifier**. The gray areas indicate classified activities.

The activity monitor was developed to apply metrics for a select type of movements, by detecting these movements based on the kinematic data. The activity monitor has two detection algorithms as shown in Figure 4. The first algorithm contains a posture detection (sitting or standing) and walking detection (cyclical or variable walking). The second algorithm contains an arm movement and reaching detection. Using a moving time window of 1 s, the type of posture is estimated. First, the classifier estimates if the patient is seated by evaluating both knee angles. If the flexion of both knees is more than 40° for the entire second, it is concluded that the patient is seated. If the participant is not seated, the feet movement will be evaluated. Foot movement can be detected by applying a step detection algorithm and using accelerometer and gyroscope signals from the sensor on both feet (Skog et al., 2010). If detected, it is inferred that the patient is walking, and if not, the patient is standing still. If walking is detected, a distinction is made between variable walking and cyclical walking (with exclusion of start and stop moments). Cyclical walking consists of at least three consecutive, alternating steps of the left and the right foot, while other types of feet movements are classified as variable walking.

Arm movement is detected by evaluating the three-dimensional hand movements relative to the pelvis. When the participant is seated and the three-dimensional hand displacement is more than 15 cm within a second (i.e., the average hand speed of at least 0.15 m/s), then that particular 1-s window will be annotated as one with hand movements of the specific arm. If the patient is walking (variable or cyclical), the three-dimensional hand displacement must be more than 25 cm within a second (i.e., the average speed of at least 0.25 m/s) for it to be considered a deliberate hand movement. A hand movement is classified as a reaching movement, when a hand displacement of more than 10 cm is away from the preferred hand position (the average hand position relative to the pelvis).

To evaluate the performance of the activity monitor, two independent reviewers manually classified four example data sets. Each data set, of a duration of 5 min, includes activities of daily living. By reviewing the kinematic reconstruction of the data, as with the activity monitor, the reviewers determined for each second the posture (sitting, standing, variable walking, or cyclical walking) of the patient and if the affected and/or non-affected arm is moving. The overall agreement between the reviewers was 85.5% of the time. Of these cases where there is an agreement between both reviewers, 86.3% was also in agreement with the activity monitor.

### 2.5 Presenting Large Amounts of Data

A large amount of movement data can be collected. At the same time, our methods must present clinically relevant, usable, and interpretable data to the health professional in order to assist clinical decision making and improve the rehabilitation program. Therefore, large amounts of data have to be presented in a concise way by, for example, using descriptive statistics and data distribution methods. The resulting set of metrics contains different types of data, each presented differently to the care-professional. We can summarize the data by using overall statistics (mean and SDs), show distributions in box plots, present relative differences for the affected and non-affected side in a ratio, or visualize body movement in graphs. These methods make it possible to aggregate potentially large amounts of data.

For the lower extremities, the mean and SD of stance and swing times, and stride lengths per foot are estimated (including all strides). The ratio of the metric values between the affected and non-affected side are also presented for the number of steps, stance and swing duration, and stride length. Box plots are created for the stance and swing durations for multiple steps of each foot. A single statistic is shown for the walking distance, duration, and walking speed. Visualizations are presented for the heel height during walking. These stride profiles show the heel height and distance of each stride during cyclical walking. They are derived from the heel position measures and the stance and swing times as described in Figure 1. The three-dimensional trajectories of each stride are projected onto a two-dimensional plane for visualization by rotating the stride trajectory in such way that all strides are oriented in the same direction.

For the upper extremities, the mean and SD are calculated for all metrics during a reaching movement. The ratios between affected and non-affected arm are calculated for the elbow and shoulder range of motions, work area, maximum reaching distance, and reaching counts. Hand–pelvis trajectories during reaching, which show the norm of the three-dimensional relative hand displacement over time during reaching, and the distribution of the hand position within the transversal plane are visualized. The hand distribution plots combine spatial and temporal information of arm movements, which is useful as not only is the area a patient can cover with his hand (with respect to the pelvis) of interest but also how frequent and for how long the hand is at a certain position. By utilizing a two-dimensional histogram plot that makes it possible to visualize the distribution of patients’ hand positions during a whole measurement. The frequency the hand is at a certain position is indicated by color, where light is less frequent and dark is the most frequent position.

### 2.6 Patient Evaluation

To evaluate the metrics and to show its ability to distinguish between a stroke patient’s quality of movement while performing structured movements in clinic and performing unstructured activities of daily living, three metrics were selected for which large differences were expected. The metrics are as follows: heel height profiles, hand distribution plots, and the total work area. The data of two participants (P1 and P2) were selected, as an example to demonstrate the potential functionality of the metrics. P1 is a 35–year-old male and P2 is a 50-year-old woman. Both patients are left-side affected and have a dominant right side. The main focus for P1 was on the evaluation of lower extremity functions. The main focus for P2 was on the evaluation of upper extremity function. The study protocol is a subset of a larger protocol that was approved by the local cantonal medical and ethical committee (registered in ClinicalTrials.gov identifier: NCT02118363). The participants were recruited from the Cereneo clinic, Center for Neurology and Rehabilitation (Vitznau, Switzerland) and gave written informed consent in accordance with the declaration of Helsinki. Two measurement sessions were selected within the rehabilitation program of both patients for which large differences were observed as the rehabilitation progressed. The first session includes a clinical assessment measurement that was captured in clinic at discharge and consisted of a 10-m walk test (Wade et al., 1987) for P1 and a predefined arm task for P2. This predefined arm task consisted of several arm movements, where the patient had to reach as far as possible and to make a circle as wide as possible over a table. The second session includes a measurement session of 3 h of movement data, which was captured at the patient’s home 4 weeks after discharge. Finally, these measurement sessions were compared with each other for each patient individually.

## 3 Results

### 3.1 Activity Monitor

**Figure 5:**
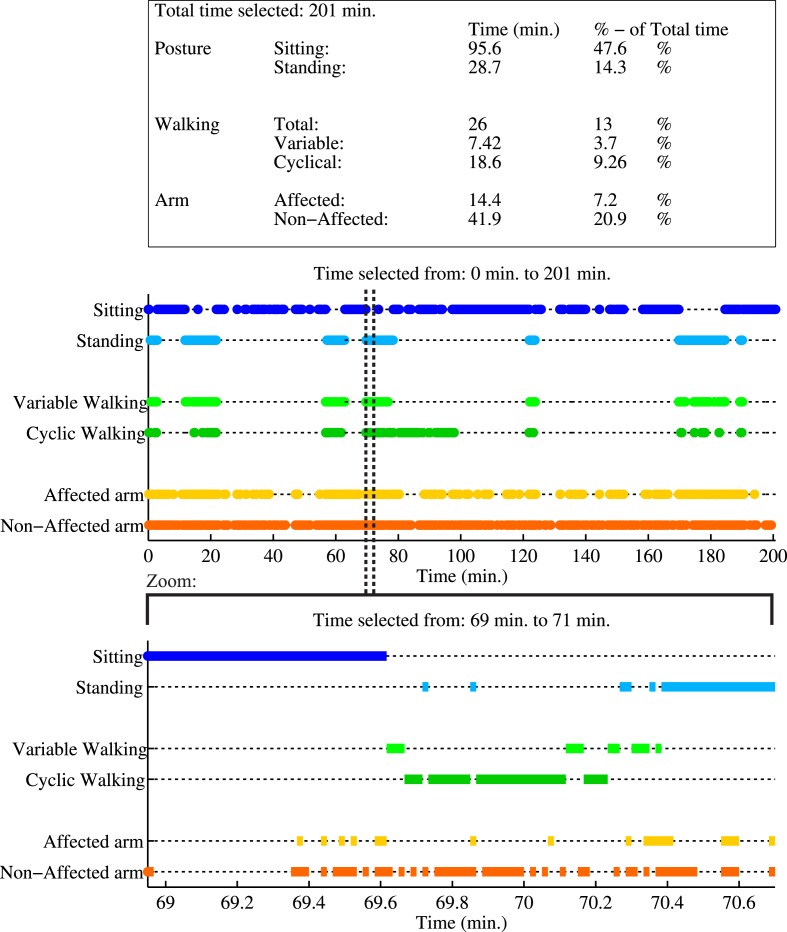
**Example report of the activity monitor of a measurement session of P2, performing activities of daily living**. First a summary report of all activities during the whole measurement is shown in the table, where the activity times are mentioned in minutes and as percentage of the total duration of the measurement session. The first graph shows the activity classification as a function of time for the whole measurement. A zoom of a selected time period is given in the lower graph. In this selection, the patient is first seated, next raises up from a chair, walks in a cyclical pattern (at least three consecutive alternating steps of both feet), and finally stands still. During this period, the non-affected arm is used more frequently than the affected arm.

In Figure 5, an example of the activity monitor and a summary report of activities are shown of P2 while performing activities of daily living at home. Each activity classification is plotted as function of time. In total, 201 min of data are analyzed, where the patient was seated for 96 min, standing for 29 min, and walking for 26 min. Data show a difference in arm usage, the non-affected arm was moving in 21% of the total time, while the affected arm was only moving 7% of the total time.

### 3.2 Lower Extremity Results of P1

**Figure 6:**
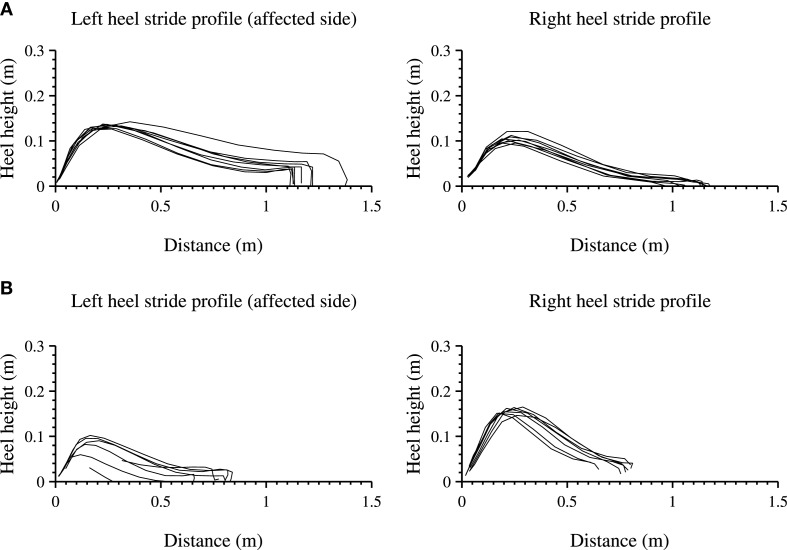
**Heel stride profiles during a structured and unstructured measurements of the left (affected side) and right foot**. Each line indicates a three-dimensional stride profile, rotated and projected onto a two-dimensional plane, starting at the origin of the graph. **(A)** shows a 10-m walk test, a structured in-clinic measurement. **(B)** shows an unstructured in-home measurement, where the patient walked in a straight line.

The lower extremities analysis of the heel height profile are presented for a 10-m walk test (10 MWT) in clinic as shown in Figure 6A and are compared with a selection where the patient is walking cyclical at home shown in Figure 6B. For each step, the heel height as function of distance (stride length) is shown for the left (affected side) and right foot.

The length of all strides for both feet of the participant are longer during the 10 MWT than during the measurement at home and the walking speed is lower 0.85 and 0.42 m/s respectively. The stride profile during the 10 MWT seems more equal for both feet as compared with the in-home measurement. In-home measurements show a stride profile that differs between the left and the right heel (non-affected side), showing a larger heel height and a steeper curve for the right heel.

### 3.3 Upper Extremity Results of P2

**Figure 7:**
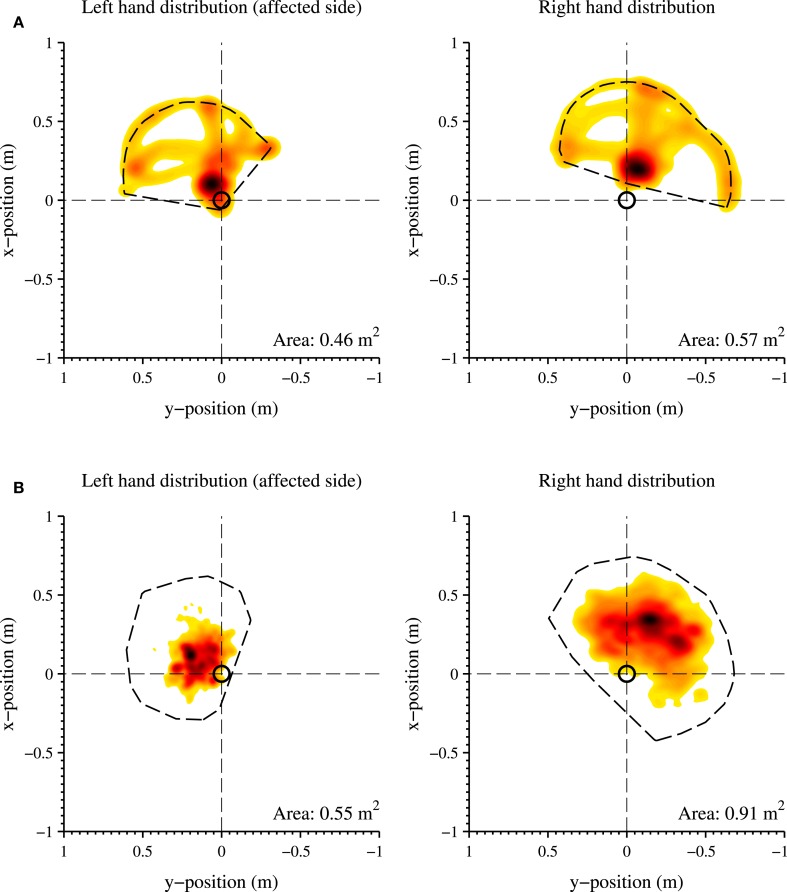
**Distribution of the hand positions relative to the pelvis (origin of the graph, indicated with a black circle) in the transversal plane, of the left (affected side) and right hand**. **(A)** Structured in-clinic measurement; **(B)** Unstructured in-home measurement. A darker color indicates a higher frequency of a specific hand position during the measurement session. A lighter color indicates a less frequent hand position at a particular location. The total work area is contoured with a dotted line and the area size is shown in the bottom right corner of each graph. The activity report of the unstructured in-home measurement is shown in Figure 5.

The distribution of hand positions relative to the pelvis is visualized for P2 in Figure 7, where each graph is divided into quadrants. The colors indicate the total time during the selected time slot at which the hand is in a certain position, where a darker color is a longer time. Figure 7A shows the predefined arm task for the affected and non-affected arm captured at discharge. Figure 7B shows a measurement session at home, while the patients perform different activities of daily living for 201 min (of which the activity report is presented in Figure 5). For both figures, the total area the patient was able to cover is shown in the lower right corner of the graph. During the predefined arm task, the patient is able to cover a larger area with the non-affected right arm (0.57 m^2^) than the left arm (0.46 m^2^). The patient was able to reach further and cover more area to the contralateral side using the non-affected right hand than with the left hand. Furthermore, P2 is able to reach more behind the pelvis with the right hand. In the home measurement, the right hand is able to cover a larger area (0.91 m^2^) than the left hand (0.55 m^2^). In these home measurements, a large difference is found in reaching movements of the right behind her pelvis compared to the left hand. The resting position of the right hand (indicated by a dark area) is more in front of the pelvis, where the left hand is closer and more along the patient’s body.

## 4 Discussion

Objective evaluation of the quality of daily life movements and intra-patient differences, using a body-worn sensing system, could be very challenging. While measuring in a daily life setting, no context on performed activities is available. Furthermore, suitable metrics are necessary to quantify relevant aspects of movement performance during daily life. Within this paper, we presented data processing methods to evaluate quality of movement. Presented methods can be used for the objective evaluation of intra-patient differences in movement quality between in-clinic measurements (more structured measurements in a controlled environment) and measurements in a daily life setting (unstructured measurements in an uncontrolled environment). The selection of metrics is based on discussions with care professionals, engineers, and researchers. To be able to make a distinction between metric values during different activities, an activity classifier was developed, which classifies different types of posture and arm activity. Finally, methods were developed to present large amounts of data in a concise manner.

As example to demonstrate potential of presented methods, data of two stroke patients were analyzed. Differences between in-clinic measurements (at the moment of discharge) and measurements during daily life (4 weeks after discharge) can be observed by applying the presented metrics and visualization methods. In the near future, any observed intra-patient progression or deterioration of movement quality could be used to decide on continuing, stopping, or adjusting rehabilitation programs. Future research should demonstrate the usability of suggested methods in more patients and how the objective information on quality of movement can be used in clinical practice.

Several limitations in the presented work should be acknowledged. First, differences between assessed movements during in-clinic measurements and measurements during daily life might be the result of the different circumstances in which the data are captured. In clinic, patients were instructed to perform a specific task that might force them to, for instance, use their affected side or walk at a specific speed. While during daily life measurements, when no specific instructions were given, it can be expected that patients will execute tasks in a most comfortable way. This may result in, e.g., a reduced usage of their affected arm, a reduced walking speed, or smaller steps. Therefore, intra-patient difference might be expected on the affected and non-affected side, however, the proposed methods can still be used to describe relative difference between their affected and non-affected side for different measurements. Second, the applied activity classifier determines only a limited number of activities (posture: sitting, standing, walking, and arm movements). This selection of activities was the result of the requirement analysis that focused on assessing quality of movements, but detection of other activities is possible as well. Previous research showed many options for identification of activities, using inertial sensors (Preece et al., 2009; Yang and Hsu, 2010). Within the presented data processing method, it is possible to replace the activity monitor filter (as in Figure 3) with a different activity classifier to identify and evaluate other activities of interest. Third, due to technical limitations, the inertial-based sensor system could only be used for measuring daily life movements up to 3 h. Within this limited period of time, no significant change in movement performance was expected. When measuring for longer periods of time, physical activity may change during one measurement session.

In addition to the presented work, future research should focus on the unobtrusiveness by reducing the number sensors, which makes the system more applicable for the assessment of daily life movements. Data of twelve inertial sensors and a full body kinematic model are used to estimate movements of all body segments. Generally, more sensors may increase the validity of activity detection and accuracy in motion analysis. Depending on the metrics of interest, sensor reduction is possible while remaining almost the same validity and accuracy (Bussmann et al., 2009). The currently used sensor system is modular, which allows the reduction of the number of sensors by using only the lower or upper part of the sensor set depending on the metrics of interest. Besides the number of sensors within this, modular part may be decreased. For instance, the number of sensors in the lower extremity module of the sensor system can be reduced to only one inertial and an additional ultrasound sensor on each foot. Using this reduced sensors system, it is already possible to estimate the same metrics that are presented in the current study (Weenk et al., 2015). Furthermore, in addition to the evaluation of movements of stroke patients, the presented data analysis methods might be useful for other group of patients, for instance, elderly people, Parkinson’s disease, multiple sclerosis, and other neurological diseases. Daily life assessment of their quality of movement might give more insight on the influence of the patient’s condition at performing activities of daily living.

## Author Contributions

FM and BK developed and tested the algorithms and drafted the manuscript. BK, JH, and AL provided the data of the stroke participants. JH, JR, JB, AL, B-JB, and PV assisted with data interpretation, helped to develop the algorithm, and helped to draft the manuscript. AL and PV supervised the research. All authors read and approved the final manuscript.

## Conflict of Interest Statement

The authors declare that the research was conducted in the absence of any commercial or financial relationships that could be construed as a potential conflict of interest.
